# ROS and Brain Gliomas: An Overview of Potential and Innovative Therapeutic Strategies

**DOI:** 10.3390/ijms17060984

**Published:** 2016-06-22

**Authors:** Mariagrazia Rinaldi, Maria Caffo, Letteria Minutoli, Herbert Marini, Rosaria Viola Abbritti, Francesco Squadrito, Vincenzo Trichilo, Andrea Valenti, Valeria Barresi, Domenica Altavilla, Marcello Passalacqua, Gerardo Caruso

**Affiliations:** 1 Department of Clinical and Experimental Medicine, University of Messina, 98125 Messina, Italy; mrinaldi@unime.it (M.R.); hrmarini@unime.it (H.M.); fsquadrito@unime.it (F.S.); vtrichilo@unime.it (V.T.); avalenti@unime.it (A.V.); 2 Department of Biomedical and Dental Sciences and Morphofunctional Imaging, Neurosurgical Clinic, University of Messina, 98125 Messina, Italy; mcaffo@unime.it (M.C.); rv.abbritti@hotmail.it (R.V.A.); daltavilla@unime.it (D.A.); mpassalacqua@unime.it (M.P.); gcaruso@unime.it (G.C.); 3 Department of Human Pathology, University of Messina, 98125 Messina, Italy; vbarresi@unime.it

**Keywords:** reactive oxygen species (ROS), glioma, tumor growth, therapeutic strategy, blood-brain barrier

## Abstract

Reactive oxygen species (ROS) represent reactive products belonging to the partial reduction of oxygen. It has been reported that ROS are involved in different signaling pathways to control cellular stability. Under normal conditions, the correct function of redox systems leads to the prevention of cell oxidative damage. When ROS exceed the antioxidant defense system, cellular stress occurs. The cellular redox impairment is strictly related to tumorigenesis. Tumor cells, through the generation of hydrogen peroxide, tend to the alteration of cell cycle phases and, finally to cancer progression. In adults, the most common form of primary malignant brain tumors is represented by gliomas. The gliomagenesis is characterized by numerous molecular processes all characterized by an altered production of growth factor receptors. The difficulty to treat brain cancer depends on several biological mechanisms such as failure of drug delivery through the blood-brain barrier, tumor response to chemotherapy, and intrinsic resistance of tumor cells. Understanding the mechanisms of ROS action could allow the formulation of new therapeutic protocols to treat brain gliomas.

## 1. Introduction

Reactive oxygen species (ROS) consist of reactive substances that derive from the partial reduction of oxygen, composed by uncoupled electrons localized in the outer shell of separate orbits. This condition makes oxygen unstable, creating free radicals through a partial reduction process. ROS can result from endogenous and exogenous sources. The former involves several mitochondrial mechanisms involving cytochrome P450 complexes metabolism, and inflammatory cascade, whereas the latter is derived from xenobiotic metabolism, ionizing radiation, heavy metals, and cigarette smoking [1,2]. Oxidative stress occurs when a mismatch between the cellular antioxidant defense system and ROS formation is established due to an increased oxidized molecule production and a decrease of their removal.

ROS have a greater reactivity and can respond quickly to similar and different molecules in human cells. Several authors reported that the interaction of ROS with organic molecules acts through the main mechanism by which oxidative stress modulates the cellular cycle [3]. Additionally, the role of reactive oxygen species in apoptosis, oncogene expression, and activation of cell signaling cascades has been widely highlighted [4]. Thus, the implication of oxidative stress has been demonstrated in promoting redox cell impairment, and finally tumorigenesis starting from different cancer cell lines [1]. In the cancer process, the increased intrinsic ROS is related to a broad spectrum of activities: stimulation of oncogenes, enhanced metabolism, and mitochondrial default [5]. Moreover, tumor cells, through the generation of free radicals, and particularly of hydrogen peroxide, may result in the damage of cells and tissues, facilitating tumor growth and invasion [6]. Additionally, various redox metals have been implicated in carcinogenesis, probably due to their capacity in creating reactive species, or metals without redox potential, or to their ability to create crucial bond with thiols [2].

Gliomas appear to be the main common primary brain tumor in the adult population [7]. A multidisciplinary management of these patients remains, to date, challenging [7]. Improvement in surgical techniques offers several benefits in terms of prolonging survival. Recently, selected targeted chemotherapies have been used in different types of tumors, even if their efficacy is still strongly debated in gliomas [8]. Factors underlying these controversial results concern the limited tumor cell drug uptake, the cell metabolism of drugs, the differential tumor response to chemotherapy, intrinsic cell resistance mechanisms, and decreased drug delivery due to the presence of the blood-brain barrier (BBB) [9].

## 2. Gliomas

Gliomas show an incidence of 45% inside the class of primary central nervous system (CNS) tumors and 77% of primary CNS malignancies [7]. Different subtypes of gliomas exist, based on the primitive originating cell line. Zulch *et al.* edited the first edition of the histological typing of tumors of the nervous system [10]. The most used classification method of these tumours, World Health Organization (WHO) classification, includes a grading scheme [11] based on the evaluation, of the surgical specimen and morphological features such as atypia, mitoses, endothelial proliferation, and necrosis. The absence of the abovementioned parameters categorizes the tumor as grade 1 composed by a slow proliferation rate, including, the most common histotype in pediatric age, pylocitic astrocytoma, pleomorphic xanthoastrocytoma, dysembryoplastic neuroepithelial tumor and ganglioglioma. Gliomas showing only atypical cells are configured as grade 2. Grade 2 gliomas are characterized by a high rate of differentiation, and a tendency of both diffuse growth into the normal brain tissue and progression into a malignant phenotype. Diffuse astrocytomas are involved in WHO grade 2. The term low-grade glioma (LGG) does not refer similarly to grade 1 astrocytomas because they differ from other gliomas with respect to their biological and genetic profile, and prognosis [12]. Patients affected by LGGs may survive for up to 20 years [13]. The growth of these tumors is continuous [14,15]; they tend to progress to higher grades, leading to neurological deterioration and finally to death [16]. Low-grade gliomas reveal a tendency to the dedifferentiation. A modern therapeutic approach for these histotypes provides for gross total resection (>90%), followed by radiotherapy and chemotherapy with temozolamide, reaching an overall slightly prolonged survival, ranging from 3 to 7 years. This variability is related to: age at diagnosis, tumor dimension, neurological impairment before therapeutic approaches and mainly to histology. Grade 3 lesions include anaplastic astrocytoma, anaplastic oligoastrocytoma and anaplastic oligodendroglioma. These demonstrate a greater cellular density, associated with a strong presence of atypia and mitoses. Grade 4 gliomas include glioblastoma and gliosarcoma. They represent the most frequent forms and the histotypes with a higher expression of malignancy. These tumors show a large amount of microvascular proliferation, pseudopalizading necrosis and a wide tendency to spread into the brain [11].

A large part of glioblastoma multiforme (GBM) (90%) are primary tumors with a multistep tumorigenesis which develops from normal glial cells. The 10% of cases are secondary neoplasms, originating through the progression from low-grade tumors (diffuse or anaplastic astrocytomas) [17]. Secondary GBMs have a decreased rate of necrosis, are diagnosed in patients with a mean age of 39 years, and present a slow growth and a better prognosis. Primary and secondary GBMs are strictly similar in terms of morphology, although the genetic patterns and the pathways leading to their development and progression are different. The main molecular and genetic features of primary GBM are represented by: amplification of epidermal growth factor receptor (EGFR), deletion or mutation of homozygous cycline dependent kinase (CDK) inhibitor p16INK4A/(CDKN2A), alterations in phosphatase and tensin homolog (PTEN) on chromosome 10, and deletion in the INK4 α [7]. TP53 mutations are common in secondary GBM, unlike the primary types. As a matter of fact, primary and secondary GBMs arise from distinct genetic pathways [18]. The most common genetic alteration of secondary GBM includes TP53 mutations and 19q loss [18,19]. Interestingly, isocitrate dehydrogenase 1 (IDH1) mutations are very frequent in secondary GBMs (>80%) and rare in primary (<5%) [20,21]. IDH1 mutations are frequent (80)% in diffuse astrocytoma WHO grade 2 and anaplastic astrocytoma WHO grade 3, in the precursor lesions of secondary glioblastomas, as well as in oligodendroglial tumors [20]. IDH2 mutations are less frequent and prevail in anaplastic oligodendrogliomas (5%) and oligoastrocytomas (6%) [20]. It has been reported that it is a genetic marker and a definitive diagnostic molecular marker of secondary glioblastomas.

### 2.1. Genetic Alterations Induced by Reactive Oxygen Species (ROS) in Carcinogenesis

Tumors arise as the result of both hereditary and somatic mutations in oncogenes and tumor suppressor genes. The amount of chromosomal damages over time leads to the abnormal survival and progressive pathological transformation of cell populations. However, the biomolecular events initiating and promoting the tumor development are not yet clarified. The carcinogenic potential induced by oxidative stress is related to the capacity of ROS to induce genotoxicity and to interfere in crucial cellular processes. Hydroxyl radicals can react with or bind bases such as pyrimidines, purines and chromatin proteins, resulting in chromosome modifications and genomic instability, and alterations in gene expression. In cancer cells, ROS accumulation could damage DNA directly, through the increase of cellular mutation and/or the enhancement of oncogenic phenotype, or indirectly acting as a secondary messenger in intracellular signaling cascades [22]. DNA alterations involving breaks of strand, purine or pyrimidine substitution and DNA-protein cross linkages, are mainly responsible for carcinogenesis process. Oxidative lesions 8-oxoGua could be a reliable biomarker of oxidative stress and of ROS-induced carcinogenesis [22]. This genomic alteration has been demonstrated in tissues with decreased concentrations of antioxidant enzymes and enhanced concentrations of ROS [23], demonstrating its essential mutagenic and carcinogenic potential. Mutations due to failed repair mechanisms are described in two tumor suppressor genes: Ras oncogene and tp53. p53 acting as a redox-active transcription factor, regulates ROS generation, mediates ROS-induced apoptotic cell death, and finally modulates cell cycle entry. It has been assumed that p53 plays a key role in the surveillance of genomic integrity [22,23] and in the apoptosis regulation, through the transcription suppression of other genes, such as c-fos, c-jun. Its mutations induce the inactivation of p16ink-4a, the super expression of CDK4 and CDK6 and of ubiquitin ligase Mdm2 and Mdm4, alterating the cell cycle regulation. Additionally, early mutation of p53 induces the dysregulation of neoangiogenesis promoting the progression from low to high-grade gliomas [24,25]. It affects vascular endothelial growth factor (VEGF) and fibroblast growth factor concentrations through the activation of corresponding gene transcription. Another effect due to the oxidative DNA stress and damage is the alterations of physiological cell repair mechanisms. In fact, according to the main reported hypotheses, the impaired cellular repair mechanisms induced by ROS oxidative stress on DNA leads to cell injury and consequently to genomic instability, mutagenesis and tumorigenesis.

### 2.2. Genetic and Molecular Alteration in Gliomas

The gliomagenesis is characterized by abnormalities in growth factor receptors. Different growth factor receptors, such as epidermal growth factor receptor (EGFR), platelet-derived growth factor receptor (PDGFR), C-Kit, vascular endhotelial growth factor receptor (VEGFR), appeared mutated in glioma cells. Indeed, PDGFR and related ligands PDGF-A and PDGF-B are increased in their expression, suggesting an autocrine or paracrine activation [26]. In GBM, several receptor tyrosine kinases (*RTK* genes), such as ERBB2, belonging to EGF receptor family, and *MET* gene, encoding hepatocyte growth factor receptor, are mutated. RTKs are implicated in cell growth and proliferation through downstream effectors such as Ras and phosphatidylinositide-3-kinase (PI3K), and are modulated by tumor suppressor genes Nf1 and phosphatase and tensin homolog (PTEN). PTEN suppresses the PI3K pathway through the dephosphorylation of phosphatidylinositol-3-4-5-triphosphate (PIP3) back to phosphatidylinositol-4-5-biphosphate [27].

Telomeres are nucleoprotein complexes implicated in the maintenance of genomic stability. At the end of chromosomes, are located several repeated nucleotides which form the telomeres and preserve the loss of encoding DNA during each duplication. In the absence of telomerase activity, telomeres shorten at each cell division. Cancer cells divide continuously because of altered telomere maintenance mechanisms. It has been reported that telomere binding proteins, α thalassemia/mental retardation syndrome X-linked (ATRX) or death-domain associated protein (DAXX) have a telomere maintenance mechanism not dependant on telomerase, and involving the promoter of the telomerase reverse transcriptase (TERT). The mutations of this gene induce an increase in telomerase expression [28]. This mutation is demonstrated in melanomas, hepatocellular carcinomas, urothelial carcinomas, primitive neuroectodermal tumors, and different subtypes of gliomas (including 83% of primary GBM).

Mutations in IDH1 and IDH2 regard the majority of low-grade gliomas in adults, defining a subtype associated with a better prognosis [20,29]. Low-grade gliomas with IDH mutation and codeletion of chromosome arms 1p and 19q (1p/19q codeletion), as oligodendrogliomas, show more effective responses to radiochemotherapy, and are associated with longer survival than diffuse gliomas, which do not report the abovementioned alterations [30]. The isocitrate dehydrogenase 1 (IDH1), a cytoplasmatic enzyme within the peroxisomes, induces the reduction of nicotinamide adenine dinucleotide phosphate (NADP+) to nicotinamide adenine dinucleotide phosphate oxidase (NADPH). A recent study demonstrated three types of lower-grade glioma disease characterized by the IDH and 1p/19q pattern. This genomic study underlined that low-grade gliomas can present an IDH mutation with a 1p/19q codeletion or a TP53 mutation, and that the majority of these tumors with wild-type IDH showed genomic and clinical similarities to primary (wild-type IDH) GBM [31]. These results suggested that low-grade glioma wild-type IDH are probably the precursors of GBM with wild-type IDH. This hypothesis is strongly supported by the fact that the median survival associated with this type of low-grade glioma is only slightly longer than the GBM wild-type IDH. Genomic analysis demonstrated that in more than 70% of patients with low or high-grade gliomas, the mutation of amino acid 132 of IDH1 occurred, and in 12% of the GBM samples [20,32]. Further studies discovered that the IDH1/2 mutations lead to the conversion, NADPH dependent, of α-KG to d-2-hydroxyglutarate (d-2HG), supporting their pro-oncogenic role [33,34]. Literature data reported that increased levels of d-2HG in cells caused oxidative stress in rat brains [35], which could potentially promote oncogenesis. In patients with gliomas, IDH1/2 mutations and TP53 mutations usually coexist [20], even if IDH2 mutations are rare. There is the possibility that cells with higher levels of 2HG may be susceptible to further malignant transformation [33].

Recently, two somatic mutations in the *H3F3A* gene, which modulates the replication-independent histone 3 variant H3.3, were identified in one-third of pediatric GBMs [36]. The H3.3 mutations are based on the amino acid substitutions at K27 or G34 [37]. Specifically, six biological subgroups of GBM, based on global DNA methylation patterns, were identified and correlated with molecular-genetic alterations and clinical parameters [37]. The 30%–40% of pediatric/young adult GBMs, are characterized by two particular epigenetic dysregulation mechanisms: the recurrent and mutually exclusive mutations in either H3F3A or IDH1, and the aberrant DNA methylation patterns. It was been demonstrated, in thalamic and midline location GBMs and in diffuse intrinsic pontine gliomas (DIPGs), a high frequency of H3F3A K27 mutations, inferring that K27 mutant GBM [35] represent an anatomically-defined subset. Non-K27 tumors, located into the hemispheres, configure the biological differences of epigenetic GBM subgroups [36]. In-depth characterization revealed that the P53, RB, and RTK pathways [32] are the three genetic pathways mainly involved in GBM, determining the enhanced cell proliferation and survival, and allowing the tumor cells to elude cell-cycle checkpoints, senescence, and apoptosis [38]. In order to confirm these genetic results, the Cancer Genoma Atlas (CGA) sequencing data provided information about somatic mutations at the level of the base pair, improving knowledge in tumor suppressors/oncogenes in GBM as well as new potential cancer encoding genes.

The deletion of NFKBIA (encoding nuclear factor of κ-light polypeptide gene enhancer in B-cells inhibitor-α), an inhibitor of the EGFR-signaling pathway, is reported to promote tumor development in GBM, which does not show mutations of EGFR [12]. Upon stimulation with a ligand such as tumor necrosis factor α (TNF-α) or lipopolysaccharide (LPS), IκBα is phosphorylated by the signalosome [39]; this phosphorylation induces the rapid degradation of IκBα, which determines the inhibition of NF-κB and leads to the translocation of p50/p65 into the nucleus to activate the transcription of downstream target genes, including cytokines, which promote tumor growth and invasiveness [39]. Interestingly, in GBM, NFKBIA deletion and EGFR amplification are mutually exclusive, raising the possibility that the two genetic events could activate the same pathway. EGFR alterations include amplifications, mutations and deletions, even though the most common is represented by the variant III deletion of the extracellular domain (EGFR-vIII mutant) [40].

The ATP-dependent helicase (*ATRX*) gene is located on chromosome Xq21.1 and modulates the integration of the histone variant H3.3 to the pericentric heterochromatin and to the telomeres [41]. ATRX has been reported to be associated with chromosomal alterations, DNA methylation, and telomeric impairment [41]. Frequently, mutations of ATRX are found in 67% of grade 2 astrocytomas, 73% of grade 3 astrocytomas, and in 57% of secondary GBMs, but rarely in primary GBM (4%) [29]. Thus, in the majority of malignant gliomas [42], the astrocyte elevated gene-1, appeared over-expressed.

## 3. ROS and Oxidative Damage

ROS derive from enzymatic reactions involving NADPH dependant oxidases, xanthine oxidase, uncoupled endothelial nitric oxide synthase, arachidonic acid, and metabolic enzymes like cytochrome P450 enzymes, lipoxygenase, and cyclooxygenase [43]. Furthermore, ROS can also be produced inside the mitochondria by the electron transport chain complexes I, II, and III, in which the electron, released from the respiratory chain, may react with molecular oxygen [44,45]. Cytosolic O^2−^ generated is converted to H_2_O_2_ by the catalytic superoxide dismutase 1 (SOD1) within the cytoplasm and the mitochondrial intermembrane space. In the presence of metal cations, such as Fe^2+^ and Cu^+^, H_2_O_2_ can be additionally reduced into OH, an extremely reactive form of ROS characterized by a strong oxidizing potential, responsible for the oxidative stress-induced cellular damage, and consequently genome instability [46]. ROS are produced in all types of cells and operate as cellular messengers essential for intra- and inter-cellular communications. There are several factors that influence the cell response to ROS damage, depending on the intensity and duration of the stimulus, and especially on the context of the signal. Low levels of ROS inside the cells play a critical role in several biological functions. ROS participate in cell progression and proliferation [47]. ROS can modify the structure of the signaling proteins through a direct process of phosphorylation, and thus they could indirectly interfere in the signal process, including immune signaling, apoptosis, metabolism, aging, and hypoxic stress [47]. Particularly, ROS can potentiate the EGF and platelet derived growth factor (PDGF) mediated signaling pathways, in which EGF and PDGF promote the tyrosine kinase activity of their receptor and subsequently their autophosphorylation, activating the related downstream pathways, such as PI3K-AKT signaling and the mitogen activated protein kinase cascade (MAPK) [46,48]. High levels of ROS, responsible for oxidative stress and of DNA and cell damage, can promote both cell survival or apoptosis in relation to the severity and the duration of exposure. DNA is strictly susceptible to exogenous and endogenous agents. ROS-induced stress could damage the DNA through a single- or double-strand breakage, base modifications, deoxyribose modification, and DNA cross-linking. A series of events like cell death, DNA mutation, replication errors, and genomic instability can occur if the oxidative damages are not repaired prior to DNA replication [1]. The majority of ROS-induced mutations involves modification of guanine, causing G → T transversions [49]. Interestingly, ROS can modify both initiation and progression of cancer [50], especially if the mutations induced involve critical genes such as oncogenes or tumor suppressor genes. As a matter of fact, oxidative stress relating factors, about one hundred oxidative DNA adducts (purine, pyrimidine, and the deoxyribose backbone), have been identified [51] in tumors, making the oxidative DNA damage the major event from which mutations can belong. The signal of mitogens begins at the external cell surface through the activation of receptor tyrosine kinases, which activate mitogen activated protein (MAP) cascades and consequently the proliferation. The result of these events is the creation of highly reactive H_2_O_2_ from different catalytic enzyme, including the NADPH oxidases [52], making the hydroxyl radical the predominant DNA ROS target [51]. The critical role of ROS in the apoptosis process has also been reported. Nuclear factor-κB, a member of Rel family transcription factors, by upregulating antiapoptotic genes [53,54], inhibits the apoptosis. In contrast, the prolonged activation of c-JunN-terminal kinase (JNK) caused by the exposure to ROS, as well as the inactivation of JNK inhibitors such as MAP kinase phosphatases, promotes apoptosis [53,54].

The brain is particularly susceptible to the damaging effects of ROS [49,55] because of its high metabolic activity and relatively decreased capacity for cellular regeneration. Protein oxidation and lipid peroxidation have been detected in the hippocampus and neocortex of patients with Alzheimer’s disease, as well as within the motor neurons in amyotrophic lateral sclerosis. It has been reported that ROS can cause neuron and astrocyte death through apoptosis and necrosis mechanisms. Further studies have demonstrated that 12-*O*-tetradecanoylphorbol-13-acetate (TPA) represents a strong stimulus of invasion/migration of U87 cells. In a recent paper, TPA-induced invasion/migration of U87 cells was sequentially blocked by protein kinase C (PKC) inhibitor, Go6976, COX-2 inhibitor, NS398, NADPH oxidase inhibitor, diphenyleneiodonium chloride (DPI), and the ROS scavengers, superoxide dismutase (SOD) and tempol, followed by the reduction of relative signal pathway [56]. Thus, it has been published that the stimulation of cyclooxygenase-2/prostaglandin E2 and metalloproteinase-9 by ROS-activated ERKs is involved in the invasion/migration of U87 glioma cells elicited by TPA, and antioxidative substances such as quercetin, baicalein and myricetin, leading to the effective suppression of invasion/migration events in glioma cells [56].

## 4. ROS Implications in the Treatment of Gliomas

ROS can promote, under physiological conditions, the cell proliferation activating growth-related signaling pathways [57,58]. However, different ROS can exert different biological effects [56,57], and cancer cells, due their high basal metabolic rate, are more susceptible, when compared to normal cells, to therapeutic agents targeting the cellular redox status [59,60]. Consequently, anticancer drugs that destroy malignant cells driving the formation of intracellular ROS have been recently developed [60]. Newer agents generally induce an increase in ROS levels (the so-called oxidative therapy) triggering the cell death through the apoptosis or necrosis processes, depending on the entity of oxidative damage [61]. These molecules could act as direct inhibitors of cancer and/or in sensitizing cancer cells to initial treatment; however, their use is associated with significant toxicity and side effects.

One of the main systems used by tumors to counteract ROS stimuli is the nuclear factor erythroid 2-related factor 2 (Nrf2), a transcription gene regulator that controls cellular redox homeostasis [62]. Jia and coworkers recently examined the role of Nrf2 in cancer cell proliferation in multiple glioma cell lines [63]. In this interesting study, the authors revealed that knocking down Nrf2 stunted glioma cell proliferation by ATP depletion, induced 5′ AMP-activated protein kinase activation and consequently inhibited the mammalian target of rapamycin (mTOR) pathway [64]. It has also been indicated that Nrf2 inhibits the differentiation of glioma stem cells and its silencing may promote cellular differentiation [64]. Accordingly, mTOR, a key mediator of PI3K signaling, has emerged as a crucial hallmark in glioblastoma patients, although therapeutic efforts to target Nrf2/mTOR molecular network are not yet conclusive [65,66]. Overall, a deeper knowledge of the Nfr2 pathway indicates that tumor cells can survive under high levels of ROS through the up-regulation of their antioxidant capacity, and this molecular adaptation also provides a greater capacity for drug inactivation. Therefore, the inhibition of the Nrf2 pathway to overcome chemoresistance appears promising [65].

Cannabidiol (CBD) is a cannabinoid with antitumor activity in different cancer types [66]. For this purpose, some authors demonstrated that the exposure to CBD decreases the glial stem cell viability in culture and the growth of primary glial stem cells-derived tumors *in vivo* through an increase in ROS levels [66]. In this paper, it has been postulated that the resistance to drugs was due to a higher expression of the antioxidant response system SLC7A11 and by partial activation of the Nrf2-related pathway [67]. These researchers thus suggested that the combination of a CBD treatment with the inhibition of system SLC7A11 through the use of specific modulators of ROS, might reduce glioma stem cells survival and tumor invasion [68]. These experimental observations further support the idea that a potential method to deactivate cancer cells, which are more susceptible to exogenous oxidative stress than normal cells, is promoting ROS creation through the oxidant pharmacological agents [61].

Nox4, one of the seven isoforms of the NADPH oxidase family can cause an increase in ROS production in GBM [69]. Moreover, Nox4 is essential for glioblastoma invasion and angiogenesis [69]. A paper by Li *et al.* suggested that inhibition of Nox4 by lentivirus-mediated small hairpin RNA could represent an attractive therapeutic option to overcome radioresistance in glioblastoma [69]. Then, in this specific condition, it could be helpful reduce ROS levels to suppress glioblastoma cell as well to increase their radiosensitivity.

Parthanatos represents programmed cell death that is regulated by hyper-activated Poly (ADP-ribose) (PAR) polymerase 1 (PARP-1), and represents a further promising approach to kill cancer cells [70]. As a matter of fact, it has been shown that glioma cells could be destroyed by Deoxypodophyllotoxin (DPT), a naturally occurring flavolignan, in a ROS-mediated PARP-1 dependent manner [71].

Other well-known flavonoids such as quercetin, catechins and proanthocyanidins protect glial cells from oxidative stress, excitotoxicity and neuroinflammation [72]. A timely review of Vidak and coworkers described the effects of main flavonoids derived from diet elements on GBM cells [72]. According to recent literature, these authors indicate that the decrease in cellular stress is used by quercetin, epigallocatechin gallate and proanthocyanidins to protect glial cells, although an increase in free radicals could also represent a potential mechanism by which proanthocyanidins for example, decrease the proliferation of glioblastoma cells and induce their death [72].

Gallic acid (GA) has shown promising results as a new anticancer agent [73,74]. Indeed, it has been reported that GA can modulate the microRNAs expression targeting the genes for some antioxidant mitochondrial enzymes in the T98G human glioblastoma cell line [75]. Experimental data have shown that different concentrations of gallic acid can cause protective or toxic effects, suggesting the need to reach an optimal concentration of this flavonoid to effectively neutralize GBM cells [75].

EGFR is also crucial for normal cell growth and differentiation [76]. However, EGFR-targeted therapies have shown limited efficacy in clinical settings [76]. A recent study revealed that the over-expression of the oncogenic variant EGFRvIII causes increased levels of ROS and a high tendency of alterations in the genome of GBM cells [77]. These data provide new knowledge on the biology of glioblastoma, confirming the crucial cross-talk between oxidative stress and PARP-1. Specifically, the ROS accumulation secondary to EGFR hyper-activation requires an increased cellular dependence by PARP-1 mediated Base Excision Repair genes [78] and it could be helpful for the development of even more effective anticancer drug combinations. Another growth factor, VEGF, stimulates vessel proliferation and it is closely related to the biology of GBM [79]. Indeed, a paper by Neurath *et al.* reported that a prolonged hypoxia promotes the expression and the activation of adenosine monophosphate-activated protein kinase (AMPK) α 2 isoform and VEGF production in glioma cell lines and GBM [80]. These investigations suggest that the suppression of VEGF signaling at different molecular steps, also involving ROS production, could eventually represent a reliable strategy in therapeutic management of GBM [81,82,83].

Bevacizumab, a recombinant monoclonal antibody, inhibiting vascular endothelial cell proliferation and angiogenesis, has been considered for treatment of recurrent glioblastoma [84]. However, this anti-angiogenic potential is related to a diffuse pattern of recurring tumors and, in this context, Src family kinase could represent a crucial hallmark [84]. Intriguingly, this molecular pathway can be activated in a mitochondrial ROS-dependent manner [85].

Overall, it is very complicated and difficult to therapeutically act on glioma cells, also considering the number of signaling pathways involved. It is likely that a better understanding at a molecular level of redox status of glioma, may open the discovery of appropriate strategies to selectively modulate ROS in this type of cancer. In light of the current knowledge about the biology of gliomas, it also appears that the more reliable antitumor strategy is making cancer cells more susceptible to pharmacological treatment overcoming the chemoresistance in glioma; indeed, recent experimental and clinical evidence suggests that the increase in ROS production above a critical threshold for the survival of cancer cells and/or the inhibition of cellular antioxidant enzymes [61] might represent a new direction to introduce innovative opportunities for anticancer drugs discovery and development.

## 5. ROS, Drug Delivery and Drug Resistance

Drugs are usually administered via the intravenous route but the blood-brain barrier (BBB) often impedes the movement of intravenously delivered drugs into the intracranial compartment [86]. In addition, brain tissue is highly sensitive, so only limited doses of therapeutic agents can be used. Large-molecule drugs and the major part of small-molecule drugs minimally cross the blood-brain barrier, except through the leakage in the areas of eventual disruptions [87]. Drug delivery is the process, involving thousands of molecules, through which a bioactive agent is released at a specific rate in a specific site. Hence, the delivery of a drug to the site of its specific action represents one of the main limits in the field of chemotherapeutic research. The application of nanotechnology to avoid these limitations represents a revolution in neuro-oncology [88]. Nanomedicine devices are ideal to deliver specific drugs directed to a selected site on a tumor because they act as carriers using a variety of processes including encapsulation, adsorption, and covalent linkage, to target cancer [89]. In order to target tumors and reduce side effects, conjugates of polymer or micellar drugs were prepared by using polyethylene glycol, enhancing the permeability and the retention effect for tumor-selective drug delivery [90]. Thus, these macromolecules showed higher pharmacokinetic properties including a longer *in vivo* half-life, a marked tumor accumulation, and finally remarkable antitumor effects. Novel biotherapeutic approaches, aiming to improve immune system defences are nowadays under investigation. Macrophages, T lymphocytes and granulocytes demonstrated a promising role for the purpose of targeted immune therapies, partially involving ROS production [91].

It has been previously reported that overproduction of ROS products is detected in different types of cancers. Therefore, the realization of nano-sized drug delivery systems specific for ROS targets could efficiently and rapidly maximize the dose of chemotherapies that target tumors in a selective way. In recent years, with the view to develop ROS-responsive biomaterials, a variety of ROS-responsive drug delivery systems have been designed to deliver anticancer agents to tumors such as drugs, proteins and small interfering RNAs (siRNAs) [92,93]. H_2_O_2_ is related to apoptosis, dysregulation of cell proliferation and DNA mutations and, thus, represents a common marker for oxidative stress in carcinogenesis [94]. The ROS involvement in cell signals and disease progression has motivated the construction of chemical ROS-responsive micro- and nanoparticels (NPs) drug carriers. The capacity to create a triggered NP carrier response in a ROS potentiated microenvironment appears interesting for the targeted drug delivery toward tumors. NPs specifically release their drug cargo guided by ROS concentration, which is commonly enhanced in the cellular environment of specific tumors, and thus show a marked cytotoxicity for cancer cells compared to non-ROS responsive molecules. It has been shown recently that, fluorescent polymer NPs bearing pinacol-type boronic ester linkers, initiate self-immolative polymer degradation and subsequently the release of the cargo drug in the presence of higher ROS concentrations, typically present in cytoplasmatic environment tumor cells [95]. However, although these ROS-responsive drug delivery systems demonstrated encouraging results in cancer therapy, their clinical application appears complex, due to some difficulties regarding safety, biodegradability, biocompatibility, toxic and side effects of the materials used. The major cancer chemotherapy failure is represented by drug resistance. Currently, chemotherapy has notably improved survival of patients with cancers, in single or in combination. The ability to elude chemotherapies is intrinsic to cancer cells. The acquisition of anticancer drug resistance includes enhanced expression of transporters that increase anticancer drugs efflux, alterations in drug metabolism, mutations in drug targets and the activation or inactivation of downstream proliferation or death signaling pathways. Various mechanisms of drug resistance have been proposed to avoid drug resistance. They can be generally categorized as affecting the pharmacodynamics or pharmacokinetics of anticancer drugs [96,97]. Pharmacodynamic resistance regards altered drug target sensitivity, enhanced DNA repair, and decreased ability to produce an apoptotic response. Pharmacokinetic resistance usually involves alterations in the stability, metabolism, excretion and distribution of chemotherapeutic drugs at the tumor site. Several tumors show high levels of ROS-derived free radicals favoring tumor progression and development. Moreover, oxidative stress modulates the efficacy of cancer treatments in different ways, including chemosensitivity, apoptosis, angiogenesis, metastasis and inflammatory responses [98]. Based on the observations of an increased GSH-redox cycling capacity in P-glycoprotein (P-gp)-over-expressing multiple drug resistance (MDR) cells, Kramer *et al.* [99] advanced the theory of dual mechanism of drug resistance, which demonstrates that the MDR should be broadened to include increased antioxidant defences in addition to the typical enhanced drug efflux mediated by P-gp pump. This theory actually has postulated the role of ROS in the development and/or the modulation of drug resistance to chemotherapies, in particular in solid tumors, confering hypoxia in the central region of tissues, angiogenesis and reperfusion.

Some tumor cells can overcome drug-induced oxidative stress by enhancing their antioxidant systems, with the outcome that a new redox balance with a more higher ROS level is established, the process of redox resetting [100]. Such drug-induced redox resetting has recently been shown to result in drug resistance. Increased levels of reduced glutathione lead to elevated chemotherapeutic drug resistance in numerous cancers [99,101]. Redox resetting has been implicated in drug resistance at multiple levels, including elevated drug efflux, altered drug metabolism and mutated drug targets [102]. In addition, ROS-induced survival signaling pathways, and the inactivation of downstream death signaling pathways, can result in drug resistance [97]. Cancer cells are capable of maintaining an oxidation-reduction reaction (redox) homeostasis state by upregulating ROS scavenging enzymes, which can confer drug resistance. Superoxide dismutase (SOD), catalase, glutathione peroxidase, and peroxiredoxin are major intracellular ROS-scavenging enzymes [103]. Furthermore, accumulating evidence indicates that elevated activity of SOD and catalase is associated with promoting cancer cell resistance to anticancer agents [103,104].

## 6. Conclusions

Malignant gliomas are still burdened by a high rate of morbidity and mortality. Conventional treatment protocols provide the gross total removal associated with several cycles of radio and chemotherapy [83,105]. Although considerable improvements in terms of surgical approaches have been reached [106], the availability of new instruments, including operative microscopes and image guided surgery, and patient survival, has only slightly increased. Gliomas present an intensive proliferation and an abnormal formation of vascular structures. These factors are probably responsible for the frequent relapses and for resistance to standard treatments. An optimal therapeutic strategy requires the study of markers specifically targeting tumors, and the application of such methodologies to deliver drugs crossing the brain blood barrier.

Oxidative stress, environmentally related factors, and carcinogenesis are closely linked. Oxygen and nitrogen reactive intermediates may directly damage DNA, or may alter its repair systems. These molecules can react with proteins, carbohydrates and lipids, and the resulting products inducing an imbalance in redox homeostasis, and may alter DNA. The main linking substances able to relate inflammation to tumorigenesis, through the oxidative/nitrosative stress, are prostaglandins and cytokines [107]. ROS may be involved in the multistep oncogenesis process at various different phases related to tumor initiation and progression, ROS-related mechanisms during tumor promotion, maintenance of the transformed state through extracellular superoxide anion generation by NOX1, and resistance to intercellular oxidative stress signals through the expression of membrane-associated catalase.

**Figure 1:**
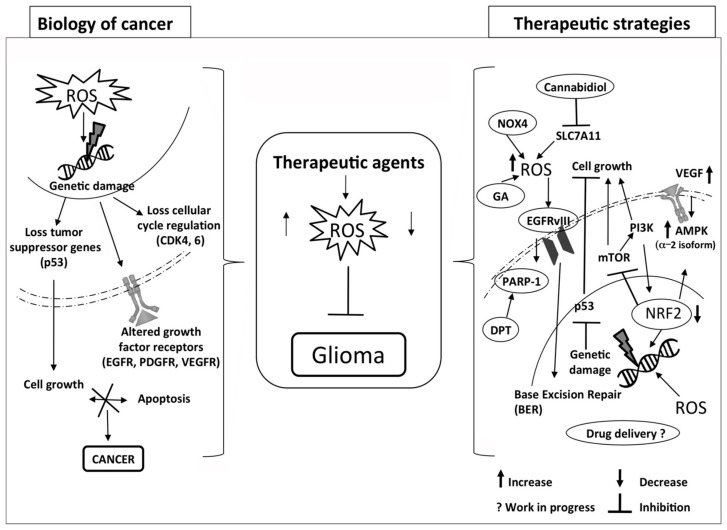
Proposal of therapeutic strategies to selectively modulate ROS pathways in glioma.

The generation of ROS can be exploited therapeutically in the treatment of cancer (Figure 1). Many dysregulating signaling modulators are been found to be associated with an increase in ROS levels, and a large number of studies reported that there are several agents able to induce ROS activation in cancer. In consideration of its cytotoxic power, ROS can also be applied to inactivate cancer cells through their selective modulation against tumors. In order to achieve this aim, a unique therapeutic strategy was developed named as “oxidation therapy”. It consists of guiding and delivering cytotoxic ROS directly to the solid tumor, or inactivating the antioxidative enzyme system [90].

The progress in new sciences such as genomics, metabonomics, proteomics and systems biology provides the background to realize and assess chemical and pharmaceutical responses to oxidative stress. Basic knowledge of cell biology and cancer biology are necessary for the rational and modern design of new and more effective therapeutic approaches for glioma treatment [108]. The targeted therapies must take into account the complex network of physiological and pathological pathways in which the redox balance is involved to develop effective drugs.
